# Aflatoxin B_1_ Exposure Aggravates Neurobehavioral Deficits and Immune Dysfunctions of Th1, Th9, Th17, Th22, and T Regulatory Cell-Related Transcription Factor Signaling in the BTBR T^+^Itpr3^tf^/J Mouse Model of Autism

**DOI:** 10.3390/brainsci13111519

**Published:** 2023-10-27

**Authors:** Mohammad Y. Alwetaid, Taghreed N. Almanaa, Saleh A. Bakheet, Mushtaq A. Ansari, Ahmed Nadeem, Sabry M. Attia, Marwa H. Hussein, Sheikh F. Ahmad

**Affiliations:** 1 Department of Botany and Microbiology, College of Science, King Saud University, Riyadh 11451, Saudi Arabia; mywetaid@hotmail.com (M.Y.A.); talmanaa@ksu.edu.sa (T.N.A.); 2 Department of Pharmacology and Toxicology, College of Pharmacy, King Saud University, Riyadh 11451, Saudi Arabia; sbakheet@ksu.edu.sa (S.A.B.); muansari@ksu.edu.sa (M.A.A.); anadeem@ksu.edu.sa (A.N.); attiasm@ksu.edu.sa (S.M.A.); mhussein3.c@ksu.edu.sa (M.H.H.)

**Keywords:** autism spectrum disorder, Aflatoxin B_1_, T cells, CD4 cells, BTBR mice, C57 mice

## Abstract

Autism spectrum disorder (ASD) is a neurodevelopmental disease characterized by impaired communication, reciprocal social interactions, restricted sociability deficits, and stereotyped behavioral patterns. Environmental factors and genetic susceptibility have been implicated in an increased risk of ASD. Aflatoxin B_1_ (AFB_1_) is a typical contaminant of food and feed that causes severe immune dysfunction in humans and animals. Nevertheless, the impact of ASD on behavioral and immunological responses has not been thoroughly examined. To investigate this phenomenon, we subjected BTBR T^+^Itpr3^tf^/J (BTBR) mice to AFB_1_ and evaluated their marble-burying and self-grooming behaviors and their sociability. The exposure to AFB_1_ resulted in a notable escalation in marble-burying and self-grooming activities while concurrently leading to a decline in social contacts. In addition, we investigated the potential molecular mechanisms that underlie the impact of AFB_1_ on the production of Th1 (IFN-γ, STAT1, and T-bet), Th9 (IL-9 and IRF4), Th17 (IL-17A, IL-21, RORγT, and STAT3), Th22 (IL-22, AhR, and TNF-α), and T regulatory (Treg) (IL-10, TGF-β1, and FoxP3) cells in the spleen. This was achieved using RT-PCR and Western blot analyses to assess mRNA and protein expression in brain tissue. The exposure to AFB_1_ resulted in a significant upregulation of various immune-related factors, including IFN-γ, STAT1, T-bet, IL-9, IRF4, IL-17A, IL-21, RORγ, STAT3, IL-22, AhR, and TNF-α in BTBR mice. Conversely, the production of IL-10, TGF-β1, and FoxP3 by CD4^+^ T cells was observed to be downregulated. Exposure to AFB_1_ demonstrated a notable rise in Th1/Th9/Th22/Th17 levels and a decrease in mRNA and protein expression of Treg. The results above underscore the significance of AFB_1_ exposure in intensifying neurobehavioral and immunological abnormalities in BTBR mice, hence indicating the necessity for a more comprehensive investigation into the contribution of AFB_1_ to the development of ASD.

## 1. Introduction

Autism spectrum disorder (ASD) is a neurodevelopmental condition characterized by deficits in social communication, repetitive behavior, restricted sociability deficits, and stereotypical patterns [1,2]. The precise origins of ASD are still not fully understood, while extensive research has indicated that immunological dysregulation plays a crucial role in the pathophysiology of ASD [3,4]. Children diagnosed with ASD exhibit immunological imbalance profiles linked to behavioral abnormalities [5]. Additionally, they undergo persistent neuroinflammatory processes in several brain regions [6]. Previous studies have reported a correlation between increased chemokine levels and behavioral deficits in individuals with ASD [5,7]. Moreover, there is a correlation between elevated chemokine receptor expression and ASD [8]. A recent study has demonstrated that the dysregulation of signaling pathways associated with Th1, Th2, Th17, and T regulatory cell (Treg)-related transcription factors is implicated in the severity of ASD [9]. However, additional research is needed to explore the precise underlying mechanism.

Alterations in the concentrations of pro-inflammatory cytokines have been documented in the bloodstream, brain, and cerebrospinal fluid of individuals diagnosed with ASD [10,11]. Several neurodevelopmental abnormalities are related to an increased level of IFN-γ [12,13]. Previous research has demonstrated elevated expression of IFN-γ in both peripheral leukocytes and brains of mice and human individuals with ASD [14,15]. In a similar vein, it has been discovered that T-bet is involved in regulating Th1 lymphocyte differentiation and is crucial in advancing neurodevelopmental diseases [16]. T-bet has been seen to enhance the infiltration of prolymphocytes, producing IL-17A in T cells linked to neuroinflammation within the CNS [17,18]. The results of this study indicate that the activation of Th1 signaling may play a crucial role in the pathogenesis of ASD.

Interleukin-9 (IL-9) is a cytokine with pro-inflammatory properties linked to the pathogenesis of autoimmune and neuroinflammatory illnesses [19]. The involvement of IL-9 in regulating the immune system in neurodevelopmental diseases has been established [20], as evidenced by the significant expression of IL-9 in the CNS [21]. The involvement of IL-9 in T cell activation and autoimmune inflammation in the CNS has been demonstrated [22]. Interferon regulatory factor 4 (IRF4) is a member of the IRF family, functioning as a transcription factor mostly expressed in immune cells. Its role involves regulating these cells’ development and function [23,24]. According to a recent study, it has been established that IRF4 plays a crucial role in the development of IL-17-producing Th17 cells [25]. In addition, it has been demonstrated that IRF4 plays a critical role in differentiating Tc17 cells in the context of central nervous system autoimmunity [26].

The role of IL-17A in the etiology of ASD is crucial [27]. Subjects with ASD and mice exhibiting autism-like symptoms have been observed to have increased levels of IL-17A [28,29]. Previous research has demonstrated a potential association between IL-17A signaling and the manifestation of autism-like symptoms in the progeny of maternal mice subjected to immunological activation [30]. A recent study found that IL-17A had a propensity to enhance sociability in mouse models that simulate neurodevelopmental problems [31]. The RAR-related orphan receptor gamma T (RORγT) is a pivotal transcription factor in regulating Th17 cells, playing a crucial role in the pathogenesis of numerous autoimmune illnesses [32]. Additionally, RORγT has been linked to neurodegenerative processes [33]. The expression of RORγT is notably elevated in lymphoid cells that produce pro-inflammatory cytokines [34]. Furthermore, previous studies have indicated an increase in the expression of STAT3, a signaling protein associated with Th17 cells, within the hippocampus. This observation implies that STAT3 may have a role in neural function [35]. Elevated levels of STAT3 have also been observed in rat models of autism and children diagnosed with ASD [9,14]. The results of this study indicate that the therapeutic modulation of the Th17-mediated immune response could potentially offer beneficial outcomes in the treatment of ASD.

Th22 cells are an important contributor to IL-22 and have a crucial impact on several neurological illnesses by facilitating the infiltration of leukocytes into the brain [36]. Tumor necrosis factor-alpha (TNF-α) is a pivotal mediator of inflammation and exhibits increased levels in the cerebrospinal fluid of individuals diagnosed with ASD [37]. Moreover, it has been observed that individuals diagnosed with ASD exhibit increased levels of TNF-α expression, indicating a potential dysregulation in their immune response [38]. The aryl hydrocarbon receptor (AhR) is strongly linked to inflammatory responses and mediates inflammatory effects in microglia [39]. AhR also has a significant impact on brain injury [40].

Prior studies have substantiated the presence of reduced amounts of regulatory T cells (Tregs) in persons diagnosed with ASD [14,41]. Tregs play a crucial role in limiting immunological activation and preventing the occurrence of self-reactivity. The absence or insufficiency of Tregs has been linked to the development of neuroinflammatory and autoimmune illnesses [14,42]. Interleukin-10 (IL-10) is a highly effective cytokine with notable anti-inflammatory properties, which holds considerable importance in ASD [43,44].

Aflatoxin B_1_ (AFB_1_) is a common contaminant found in food and feed, posing significant health risks for humans and animals [45]. AFB_1_ undergoes hepatic metabolism, generating reactive metabolites that induce growth inhibition, starvation, and immune system modifications [46]. The toxicity induced by AFB_1_ has been documented in various organs, including the pancreas, bladder, kidneys, and CNS [47]. AFB_1_ has been observed to induce disruption of the blood–brain barrier through its toxic effects on vascular endothelial cells and astrocytes [48,49]. Several investigations have demonstrated that AFB_1_ can stimulate the release of inflammatory mediators in the CNS, resulting in immunotoxicity and neurodegeneration [50,51].

The BTBR T^+^Itpr3^tf/^J (BTBR) mouse strain, characterized by inbreeding, displays a variety of behavioral traits resembling those observed in individuals with ASD. Consequently, this strain serves as a valuable model for studying ASD. The BTBR mice demonstrate a characteristic decrease in social communication and interaction, as well as social impairments, modifications in vocalization, and increased repetitive behaviors. These observed characteristics align with the diagnostic criteria for ASD [52,53]. The presence of immune system abnormalities has been identified in BTBR mice, and a significant association exists between these abnormalities and the immunological status of children diagnosed with ASD [54,55]. The BTBR mice have elevated concentrations of pro-inflammatory mediators [56]. In a previous study, it was observed that BTBR mice displayed elevated amounts of chemokines and chemokine receptors, as well as modifications in Th1, Th9, Th17, Th22, and Treg cells [9,54,57,58]. In this work, the researchers postulated that exposure to AFB_1_ in BTBR mice is linked to neurobehavioral functioning and immunological response impairments, as seen by the worsening of behavioral deficits and immune irregularities.

## 2. Results

### 2.1. AFB_1_ Exposure Declines Social Behavior in BTBR Mice

**Figure 1:**
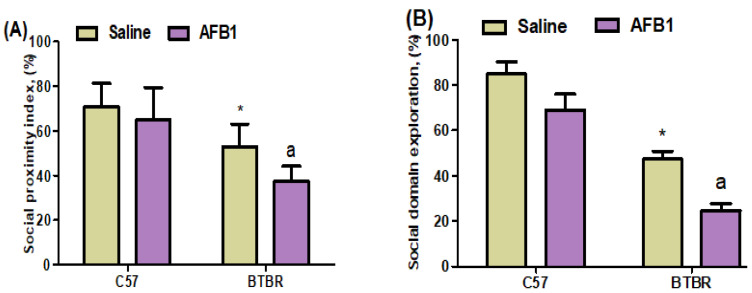
Displays two distinct entities labeled (**A**,**B**). This study aimed to investigate the impact of administering AFB_1_ on the sociability parameters test, namely social proximity index and social domain exploration, in BTBR and C57 mice. The experimental procedure involved the administration of AFB_1_ at a dosage of 1250 µg/kg/d to BTBR and C57 mice for 28 days, using the oral route of administration (p.o.). The control groups of C57 and BTBR mice were administered saline orally. The statistical analysis was performed using two-way ANOVA followed by Tukey’s post hoc test, which was corrected for multiple comparisons. All data are shown as mean ± SD (*n* = 10/group). * *p* < 0.05, compared to saline-treated C57 mice; ^a^ *p* < 0.05, compared to saline-treated BTBR mice.

To investigate the potential impact of AFB_1_ exposure on the social interactions of BTBR mice, a three-chamber social interaction test was undertaken to evaluate their sociability. The saline-treated and AFB_1_-treated C57 mice exhibited comparable durations of time spent in the experimental setup, with each chamber being about identical in size. In contrast, AFB_1_-treated BTBR mice exhibited decreased social proximity index and social domain exploration, as compared to saline-treated BTBR mice (Figure 1A,B): strain [BTBR < C57: F(1,36) = 176.9, *p* = 0.0001], exposure [AFB_1_ < no AFB_1_: F(1,36) = 74.36, *p* = 0.0001], and exposure × strain interaction [F(1,36) = 2.432, *p* = 0.0265] all exerted a significant effect on the social proximity index (Figure 1A). Similarly, strain [BTBR < C57: F(1,36) = 414.6, *p* = 0.0001], exposure [AFB_1_ < no AFB_1_: F(1,36) = 91.06, *p* = 0.0001], and exposure × strain interaction [F(1,36) = 3.154, *p* = 0.0206] significantly altered social domain exploration (Figure 1B). Thus, AFB_1_ exposure reduced the social deficits in BTBR mice during the three-chamber social test.

### 2.2. AFB_1_ Exposure Alters Repetitive Behaviors in BTBR Mice

**Figure 2:**
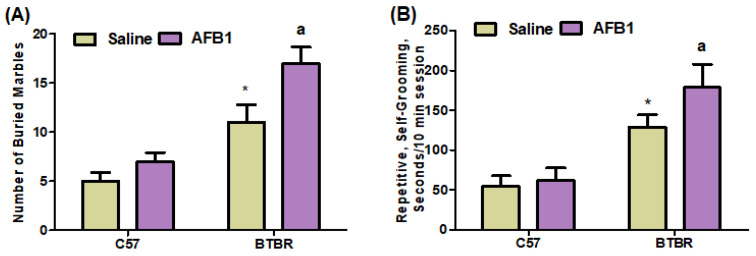
Displays two distinct components labeled (**A**,**B**). The impact of AFB1 treatment on recurrent marble-burying and self-grooming behaviors in BTBR and C57 mice. The experimental procedure involved the administration of AFB_1_ at a dosage of 1250 µg/kg/d to both BTBR and C57 mice for 28 days. The administration was carried out orally. The control groups of C57 and BTBR mice were administered saline exclusively orally. The statistical analysis was performed using two-way ANOVA followed by Tukey’s post hoc test, which was corrected for multiple comparisons. All data are shown as mean ± SD (*n* = 10/group). * *p* < 0.05, compared to saline-treated C57 mice; ^a^ *p* < 0.05, compared to saline-treated BTBR mice.

In this study, we assessed marble-burying and self-grooming behaviors in C57 and BTBR mice to analyze and evaluate the presence of repetitive behaviors. AFB_1_-treated BTBR mice showed a significant increase in the average number of marbles buried, as compared to saline-treated BTBR mice [BTBR > C57: F(1,36) = 441.9, *p* = 0.0001], exposure [AFB_1_ > no AFB_1_: F(1,36) = 87.27, *p* = 0.0001], and exposure × strain interaction [F(1,36) = 21.82, *p* = 0.0001] effects (Figure 2A). AFB_1_-treated BTBR mice also spent more time self-grooming than saline-treated BTBR mice (Figure 2B): strain [BTBR > C57: F(1,36) = 146.0, *p* = 0.0001], exposure [AFB_1_ > no AFB_1_: F(1,36) = 13.41, *p* = 0.0016], and exposure × strain interaction [F(1,36) = 7.127, *p* = 0.0147] effects. These results indicated that AFB_1_ exposure caused severe, stereotyped, and repetitive deficits in BTBR mice.

### 2.3. AFB_1_ Exposure Upregulates Th1 Cells

**Figure 3:**
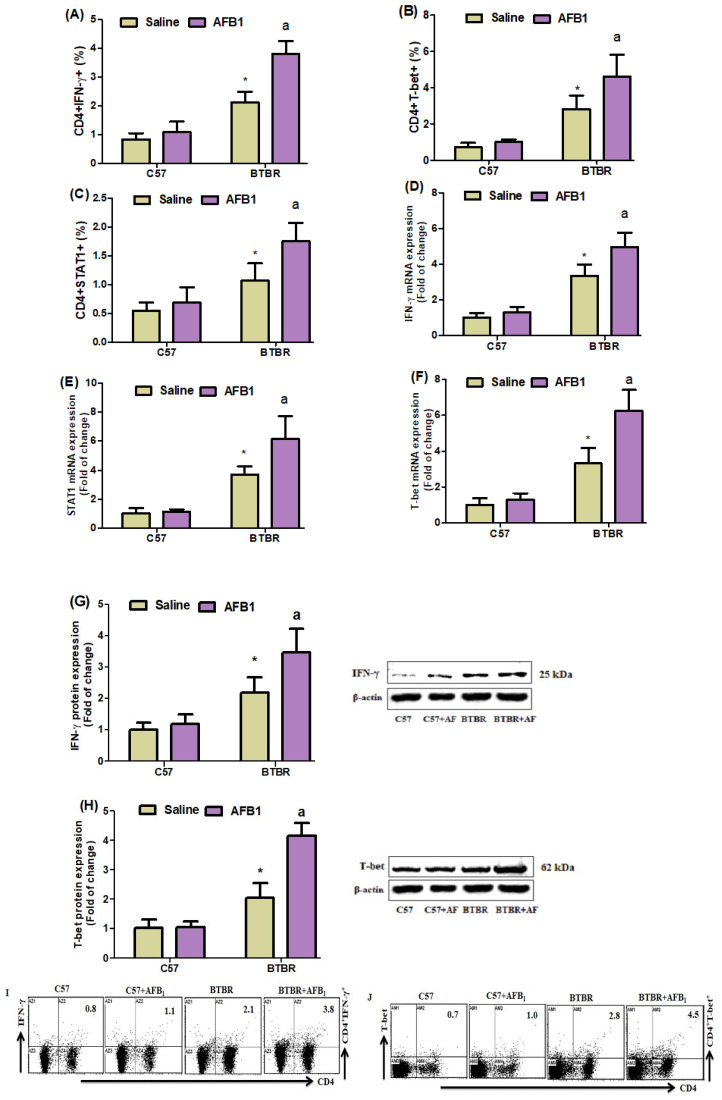
(**A**–**C**) Flow cytometry was used to examine the effect of AFB_1_ administration on IFN-γ, STAT1-, and T-bet-expressing CD4^+^ T cells in the spleen. (**D**–**F**) RT-PCR was used to examine the brain’s IFN-, STAT1, and T-bet mRNA expression. (**G**,**H**) Western blot analysis was used to examine IFN-γ and T-bet protein expression in the brain. (**I**,**J**) FSC-SSC dot plots of CD4^+^IFN-γ^+^ and CD4^+^T-bet^+^ cells from each mouse spleen cell were collected. BTBR and C57 mice were given 1250 µg/kg/d of AFB1 orally (p.o.) daily for 28 days. The statistical analysis was performed using two-way ANOVA followed by Tukey’s post hoc test, which was corrected for multiple comparisons. All data are shown as mean ± SD (*n* = 10/group). * *p* < 0.05, compared to saline-treated C57 mice; ^a^ *p* < 0.05, compared to saline-treated BTBR mice.

To examine the potential impact of AFB_1_ exposure on Th1 cells in BTBR mice, we conducted flow cytometry analyses to assess the expression of IFN-γ, STAT1, and T-bet in CD4^+^ T cells. It was observed that the BTBR mice treated with AFB_1_ exhibited notably elevated quantities of all the above cell types compared to the BTBR mice treated with saline (Figure 3A–C): strain [BTBR > C57: F(1,36) = 186.2, *p* = 0.0001], exposure [AFB_1_ > no AFB_1_: F(1,36) = 43.52, *p* = 0.0001], and exposure × strain interaction [F(1,36) = 23.77, *p* = 0.0001] effects, in the case of CD4^+^IFN-γ^+^ cells; strain [BTBR > C57: F(1,36) = 52.64, *p* = 0.0001], exposure [AFB_1_ > no AFB_1_: F(1,36) = 14.26, *p* = 0.0012], and exposure × strain interaction [F(1,36) = 6.073, *p* = 0.0229] effects, in the case of CD4^+^STAT1^+^ cells; and strain [BTBR > C57: F(1,36) = 94.12, *p* = 0.0001], exposure [AFB_1_ > no AFB_1_: F(1,36) = 12.44, *p* = 0.0021], and exposure × strain interaction [F(1,36) = 6.620, *p* = 0.0182] effects, in the case of CD4^+^T-bet^+^ cells.

To better understand the mechanism of action of AFB_1_ exposure, we used RT-PCR and Western blot studies to examine the levels of IFN-, STAT1, and T-bet mRNA and protein expression in brain tissue. AFB_1_-treated BTBR mice displayed upregulated IFN-γ mRNA, as compared to the saline-treated BTBR mice (Figure 3D–F): strain [BTBR > C57: F(1,20) = 178.2, *p* = 0.0001], exposure [AFB_1_ > no AFB_1_: F(1,20) = 17.76, *p* = 0.0004], and exposure × strain interaction [F(1,20) = 8.611, *p* = 0.0082] effects, in the case of IFN-γ; strain [BTBR > C57: F(1,20) = 117.2, *p* = 0.0001], exposure [AFB_1_ > no AFB_1_: F(1,20) = 12.98, *p* = 0.0018], and exposure × strain interaction [F(1,20) = 10.54, *p* = 0.0040] effects, in the case of STAT1; and strain [BTBR > C57: F(1,20) = 134.6, *p* = 0.0001], exposure [AFB_1_ > no AFB_1_: F(1,20) = 25.96, *p* = 0.0001], and exposure × strain interaction [F(1,20) = 17.87, *p* = 0.0004] effects, in the case of T-bet.

We then examined the IFN-γ and T-bet protein expression levels in brain tissue. AFB_1_-treated BTBR animals had higher IFN-γ and T-bet protein amounts than saline-treated BTBR mice (Figure 3G,H): strain [BTBR > C57: F(1,20) = 74.96, *p* = 0.0001], exposure [AFB_1_ > no AFB_1_: F(1,20) = 13.46, *p* = 0.0015], and exposure × strain interaction [F(1,20) = 7.622, *p* = 0.0121] effects, in the case of IFN-γ; strain [BTBR > C57: F(1,20) = 179.4, *p* = 0.0001], exposure [AFB_1_ > no AFB_1_: F(1,20) = 48.47, *p* = 0.0001], and exposure × strain interaction [F(1,20) = 45.50, *p* = 0.0001] effects, in the case of T-bet. Overall, AFB_1_ exposure significantly affected the Th1 cells in BTBR mice.

### 2.4. AFB_1_ Exposure Upregulates Th9 Cells

**Figure 4:**
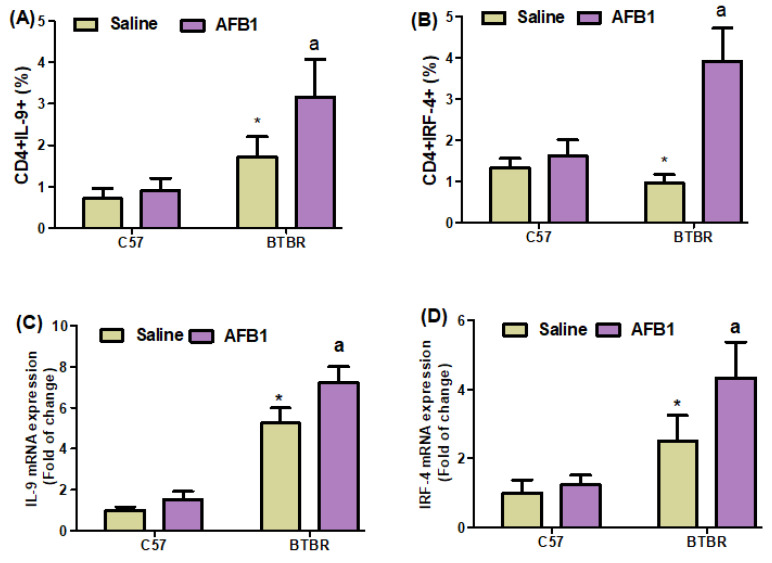

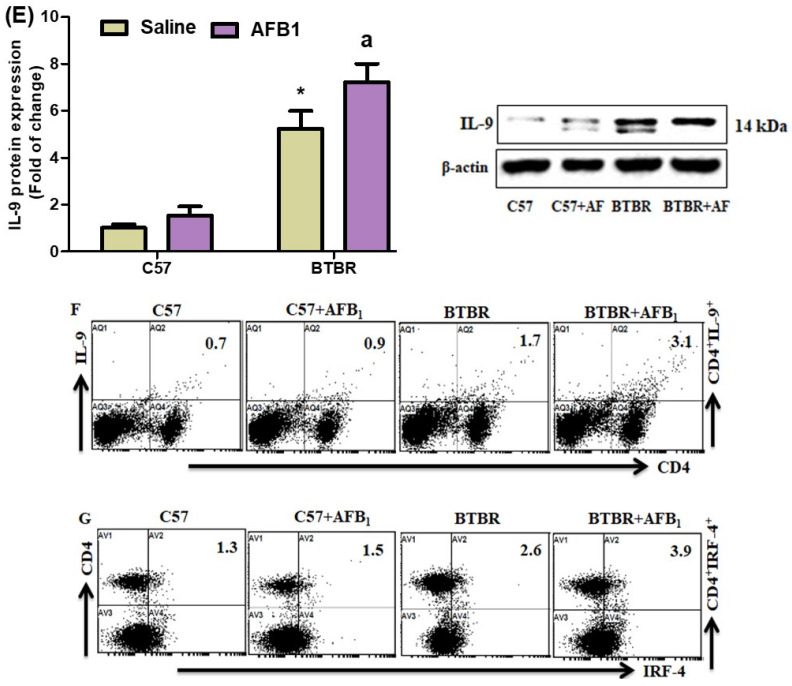
(**A**,**B**) Flow cytometry was used to examine the effect of AFB_1_ treatment on IL-9- and IRF4-expressing CD4^+^ T cells in the spleen. (**C**,**D**) RT-PCR was used to examine mRNA expression of IL-9 and IRF4 in the brain. (**E**) Western blot analysis was used to examine the protein expression of IL-9 in the brain. (**F**,**G**) FSC-SSC dot plots of CD4^+^IL-9^+^ and CD4^+^IRF4^+^ cells from each mouse spleen cell. BTBR and C57 mice were given 1250 µg/kg/d of AFB_1_ orally (p.o.) daily for 28 days. Saline was administered p.o. to the control C57 and BTBR mice. The statistical analysis was performed using two-way ANOVA followed by Tukey’s post hoc test, which was corrected for multiple comparisons. All data are shown as mean ± SD (*n* = 10/group). * *p* < 0.05, compared to saline-treated C57 mice; ^a^ *p* < 0.05, compared to saline-treated BTBR mice.

To investigate the effect of AFB_1_ exposure further, we examined Th9 cells in the spleen and brain of BTBR mice. AFB_1_-treated BTBR mice had considerably greater percentages of IL-9- and IRF4-expressing CD4^+^ T cells than saline-treated BTBR mice (Figure 4A,B): strain [BTBR > C57: F(1,20) = 53.43, *p* = 0.0001], exposure [AFB_1_ > no AFB_1_: F(1,20) = 13.26, *p* = 0.0016], and exposure × strain interaction [F(1,20) = 7.856, *p* = 0.0110] effects, in the case of CD4^+^IL-9^+^ cells; strain [BTBR > C57: F(1,36) = 25.19, *p* = 0.0001], exposure [AFB_1_ > no AFB_1_: F(1,36) = 71.91, *p* = 0.0001], and exposure × strain interaction [F(1,36) = 48.78, *p* = 0.0001] effects, in the case of CD4^+^IRF4^+^ cells.

Next, we found that AFB_1_ treatment raised the mRNA expression levels of IL-9 and IRF4 in BTBR mice compared to saline-treated BTBR mice (Figure 4C,D): strain [BTBR > C57: F(1,20) = 435.6, *p* = 0.0001], exposure [AFB_1_ > no AFB_1_: F(1,20) = 27.49, *p* = 0.0001], and exposure × strain interaction [F(1,20) = 9.247, *p* = 0.0065] effects, in the case of IL-9; strain [BTBR > C57: F(1,20) = 69.26, *p* = 0.0001], exposure [AFB_1_ > no AFB_1_: F(1,20) = 14.05, *p* = 0.0013], and exposure × strain interaction [F(1,20) = 8.253, *p* = 0.0094] effects, in the case of IRF4.

The IL-9 protein levels in AFB_1_-treated BTBR animals were considerably greater than those in saline-treated BTBR mice (Figure 4E): strain [BTBR > C57: F(1,20) = 435.6, *p* = 0.0001], exposure [AFB_1_ > no AFB_1_: F(1,20) = 27.49, *p* = 0.0001], and exposure × strain interaction [F(1,20) = 9.247, *p* = 0.0065] effects. Therefore, it may be inferred from our findings that exposure to AFB_1_ in BTBR mice enhances the expression of Th9 cells, potentially influencing the start and modulation of neurobehavioral responses.

### 2.5. AFB_1_ Exposure Increases Th17 Cells in BTBR Mice

**Figure 5:**
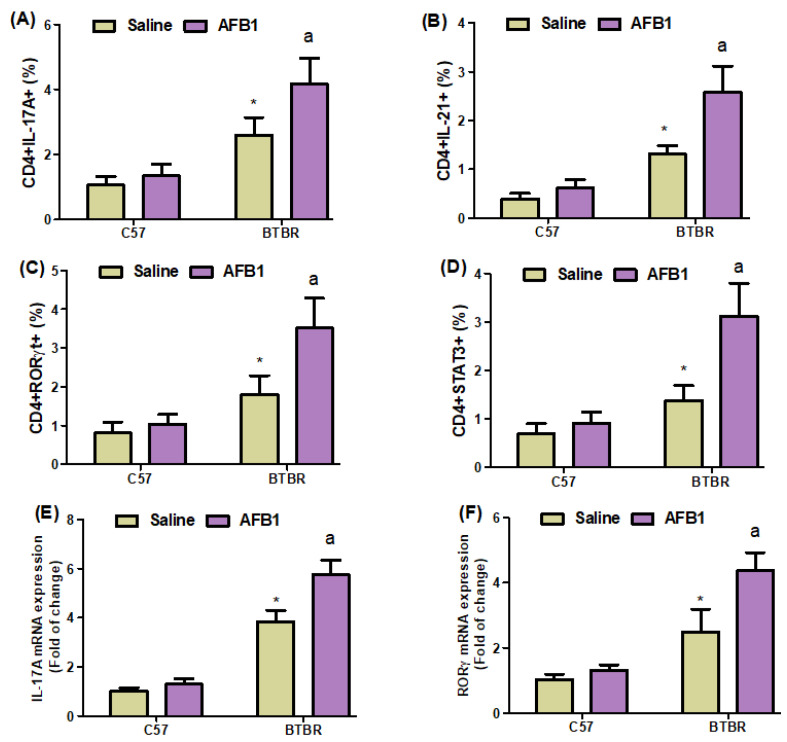

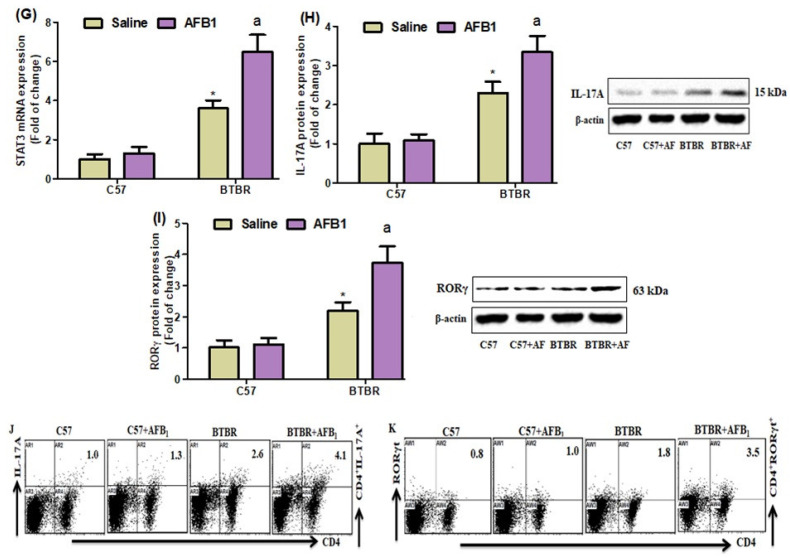
(**A**–**D**) Flow cytometry was used to examine the effect of AFB_1_ treatment on IL-17A-, IL-21-, RORγT-, and STAT3-expressing CD4^+^ T cells in the spleen. (**E**–**G**) IL-17A, RORγ, and STAT3 expression in the brain was investigated using RT-PCR. (**H**,**I**) Protein expression of IL-17A and RORγ in the brain was investigated using Western blot analysis. (**J**,**K**) Representative FSC-SSC dot plots of CD4^+^IL-17A^+^ and CD4^+^RORγT^+^ cells were taken from each mouse spleen cell. BTBR and C57 mice were treated with 1250 µg/kg/d of AFB_1_ daily for 28 d via oral administration (p.o.). The control C57 and BTBR mice received saline only via p.o. administration. The statistical analysis was performed using two-way ANOVA followed by Tukey’s post hoc test, which was corrected for multiple comparisons. All data are shown as mean ± SD (*n* = 10/group). * *p* < 0.05, compared to saline-treated C57 mice; ^a^ *p* < 0.05, compared to saline-treated BTBR mice.

Flow cytometry also demonstrated a significant increase in IL-17A-, IL-21-, RORγT-, and STAT3-expressing CD4^+^ T cells in AFB_1_-treated BTBR mice spleens compared to saline-treated BTBR mice spleens (Figure 5A–D): strain [BTBR > C57: F(1,20) = 99.87, *p* = 0.0001], exposure [AFB_1_ > no AFB_1_: F(1,20) = 18.16, *p* = 0.0004], and exposure × strain interaction [F(1,20) = 8.613, *p* = 0.0082] effects, in the case of CD4^+^IL-17A^+^ cells; strain [BTBR > C57: F(1,36) = 136.7, *p* = 0.0001], exposure [AFB_1_ > no AFB_1_: F(1,36) = 36.94, *p* = 0.0001], and exposure × strain interaction [F(1,36) = 17.92, *p* = 0.0004] effects, in the case of CD4^+^IL-21^+^ cells; strain [BTBR > C57: F(1,36) = 75.20, *p* = 0.0001], exposure [AFB_1_ > no AFB_1_: F(1,36) = 23.06, *p* = 0.0001], and exposure × strain interaction [F(1,36) = 13.57, *p* = 0.0015] effects, in the case of CD4^+^RORγT^+^ cells; and strain [BTBR > C57: F(1,20) = 77.19, *p* = 0.0001], exposure [AFB_1_ > no AFB_1_: F(1,20) = 35.10, *p* = 0.0001], and exposure × strain interaction [F(1,20) = 21.26, *p* = 0.0002] effects, in the case of CD4^+^STAT3^+^ cells.

Next, we investigated whether AFB_1_ influenced IL-17A, RORγ, and STAT3 mRNA expression in BTBR brain tissue and discovered that AFB_1_-treated BTBR mice had significantly higher IL-17A, RORγ, and STAT3 mRNA expression than saline-treated BTBR mice (Figure 5E–G): strain [BTBR > C57: F(1,20) = 512.4, *p* = 0.0001], exposure [AFB_1_ > no AFB_1_: F(1,20) = 46.13, *p* = 0.0001], and exposure × strain interaction [F(1,20) = 25.16, *p* = 0.0001] effects, in the case of IL-17A; strain [BTBR > C57: F(1,20) = 146.1, *p* = 0.0001], exposure [AFB_1_ > no AFB_1_: F(1,20) = 33.37, *p* = 0.0001], and exposure × strain interaction [F(1,20) = 18.47, *p* = 0.0004] effects, in the case of RORγ; and strain [BTBR > C57: F(1,20) = 340.4, *p* = 0.0001], exposure [AFB_1_ > no AFB_1_: F(1,20) = 57.78, *p* = 0.0001], and exposure × strain interaction [F(1,20) = 37.74, *p* = 0.0001] effects, in the case of STAT3.

IL-17A and RORγ protein expression levels in brain tissue were higher in AFB_1_-treated BTBR mice than in saline-treated BTBR mice (Figure 5H,I): strain [BTBR > C57: F(1,20) = 218.4, *p* = 0.0001], exposure [AFB_1_ > no AFB_1_: F(1,20) = 22.51, *p* = 0.0001], and exposure × strain interaction [F(1,20) = 15.73, *p* = 0.0008] effects, in the case of IL-17A; strain [BTBR > C57: F(1,20) = 191.0, *p* = 0.0001], exposure [AFB_1_ > no AFB_1_: F(1,20) = 35.94, *p* = 0.0001], and exposure × strain interaction [F(1,20) = 27.71, *p* = 0.0001] effects, in the case of RORγ. These results suggested that AFB_1_ exposure exerts pro-inflammatory effects in BTBR mice.

### 2.6. AFB_1_ Exposure Upregulates Th22 Expression

**Figure 6:**
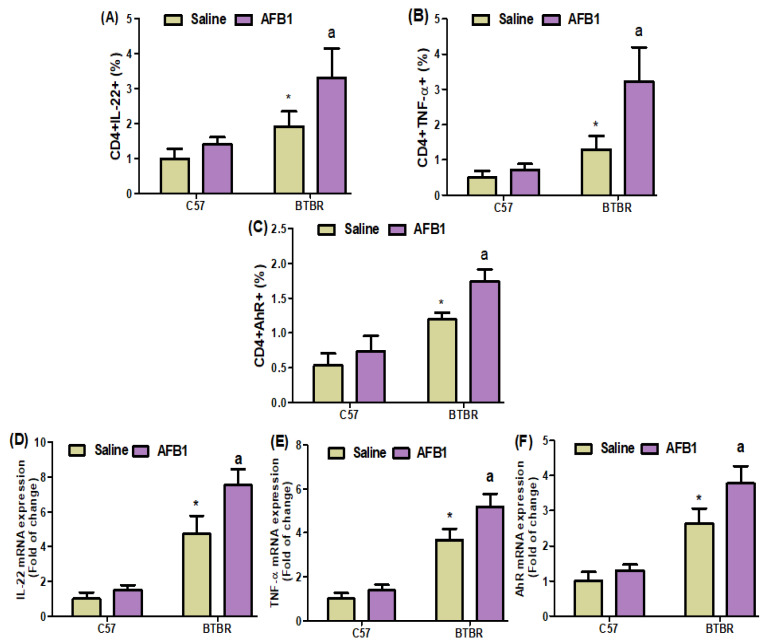

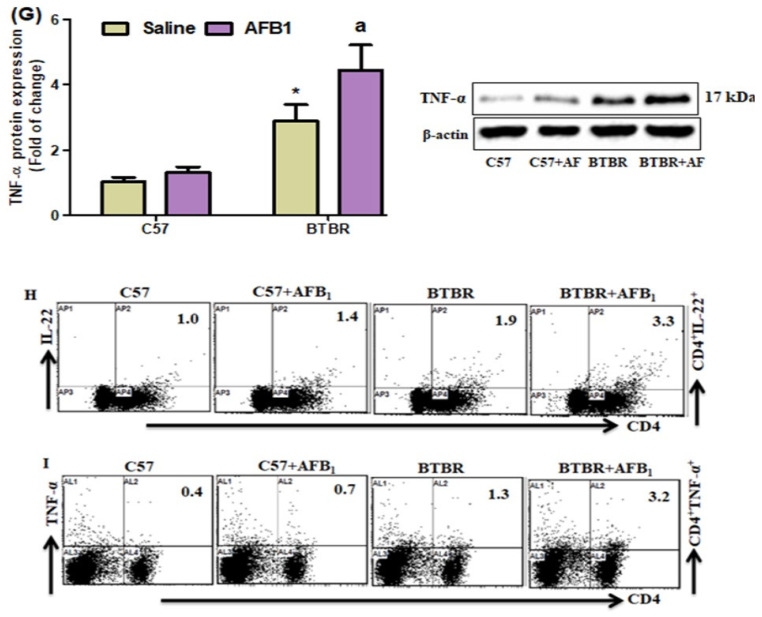
(**A**–**C**) Flow cytometry was used to examine the effect of AFB1 treatment on IL-22-, TNF-α, and AhR-expressing CD4^+^ T cells in the spleen. (**D**–**F**) RT-PCR was used to examine the brain’s mRNA expression of IL-22, TNF-α, and AhR. (**G**) Protein expression of TNF-α in the brain was investigated using Western blot analysis. (**H**,**I**) Representative FSC-SSC dot plots of CD4^+^IL-22^+^ and CD4^+^AhR^+^ cells were taken from each mouse spleen cell. BTBR and C57 mice were treated with 1250 µg/kg/d of AFB_1_ daily for 28 d via oral administration (p.o.). The control C57 and BTBR mice received saline only via p.o. administration. The statistical analysis was performed using two-way ANOVA followed by Tukey’s post hoc test, which was corrected for multiple comparisons. All data are shown as mean ± SD (*n* = 10/group). * *p* < 0.05, compared to saline-treated C57 mice; ^a^ *p* < 0.05, compared to saline-treated BTBR mice.

Flow cytometric analyses of spleen cells revealed a substantial increase in IL-22-, AhR-, and TNF-α-expressing CD4^+^ T cells in the AFB_1_-treated BTBR mice, as compared to those in the saline-treated BTBR mice (Figure 6A–C): strain [BTBR > C57: F(1,20) = 47.86, *p* = 0.0001], exposure [AFB_1_ > no AFB_1_: F(1,20) = 19.37, *p* = 0.0003], and exposure × strain interaction [F(1,20) = 5.674, *p* = 0.0273] effects, in the case of CD4^+^IL-22^+^ cells; strain [BTBR > C57: F(1,36) = 142.7, *p* = 0.0001], exposure [AFB_1_ > no AFB_1_: F(1,36) = 29.15, *p* = 0.0001], and exposure × strain interaction [F(1,36) = 6.172, *p* = 0.0220] effects, in the case of CD4^+^AhR^+^ cells; and strain [BTBR > C57: F(1,36) = 57.58, *p* = 0.0001], exposure [AFB_1_ > no AFB_1_: F(1,36) = 23.99, *p* = 0.0001], and exposure × strain interaction [F(1,36) = 15.26, *p* = 0.0009] effects, in the case of CD4^+^TNF-α^+^ cells.

AFB_1_ exposure in BTBR mice increased the IL-22, TNF-α, and AhR mRNA expression levels, as compared to those observed in the saline-treated BTBR mice (Figure 6D–F): strain [BTBR > C57: F(1,20) = 262.8, *p* = 0.0001], exposure [AFB_1_ > no AFB_1_: F(1,20) = 30.33, *p* = 0.0001], and exposure × strain interaction [F(1,20) = 14.92, *p* = 0.0010] effects, in the case of IL-22; strain [BTBR > C57: F(1,20) = 330.8, *p* = 0.0001], exposure [AFB_1_ > no AFB_1_: F(1,20) = 28.33, *p* = 0.0001], and exposure × strain interaction [F(1,20) = 9.892, *p* = 0.0051] effects, in the case of TNF-α; and strain [BTBR > C57: F(1,20) = 196.7, *p* = 0.0001], exposure [AFB_1_ > no AFB_1_: F(1,20) = 24.10, *p* = 0.0001], and exposure × strain interaction [F(1,20) = 8.267, *p* = 0.0094] effects, in the case of AhR.

Upon evaluating the effects of AFB_1_ exposure on TNF-α protein level, the level was found to be significantly higher in the AFB_1_-treated BTBR mice than in the saline-treated BTBR mice (Figure 6G): strain [BTBR > C57: F(1,20) = 166.2, *p* = 0.0001], exposure [AFB_1_ > no AFB_1_: F(1,20) = 23.28, *p* = 0.0001], and exposure × strain interaction [F(1,20) = 10.97, *p* = 0.0035] effects, in the case of TNF-α. Thus, AFB_1_ seemed to increase the number of Th22 cells, suggesting that abnormal immune responses in ASD may influence neural development.

### 2.7. AFB_1_ Exposure Decreases Treg (IL-10, TGF-Β1, and Foxp3) Expression in BTBR Mice

**Figure 7:**
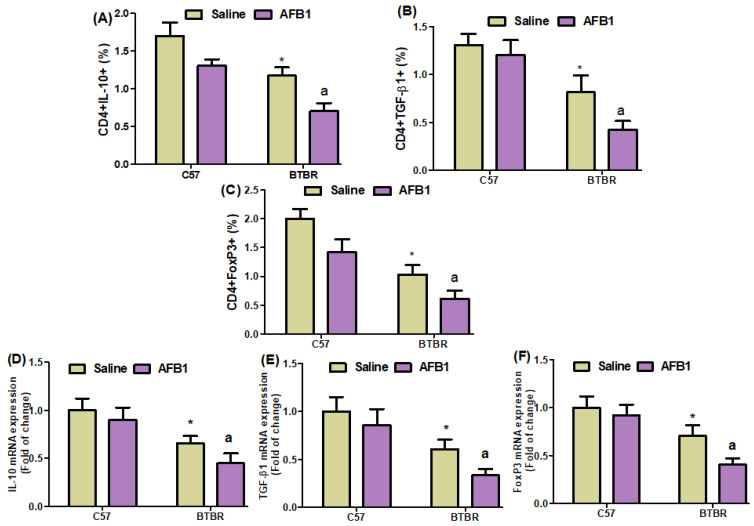

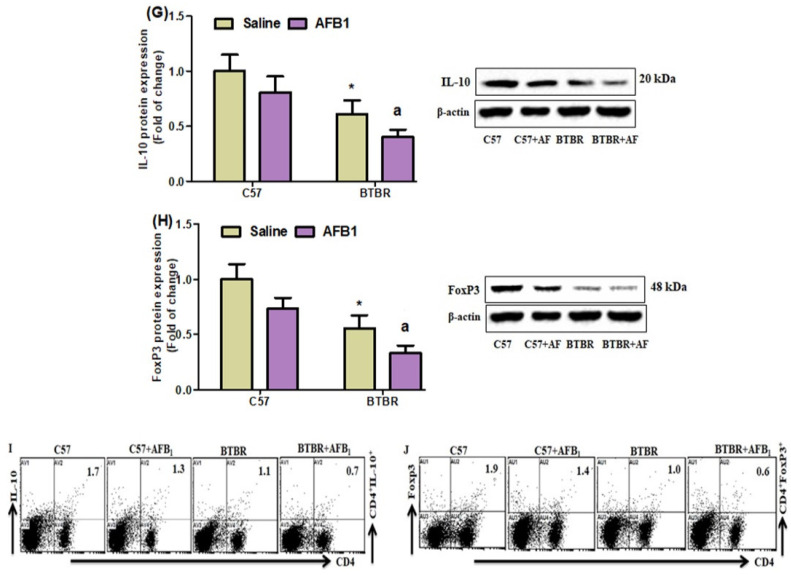
(**A**–**C**) Flow cytometry was used to examine the effect of AFB1 treatment on IL-10-, TGF-β1-, and FoxP3-expressing CD4^+^ T cells in the spleen. (**D**–**F**) mRNA expression levels of IL-10, TGF-β1, and FoxP3 in the brain were investigated using RT-PCR. (**G**,**H**) Protein expression of IL-10 and FoxP3 in the brain was investigated using Western blot analysis. (**I**,**J**) Representative FSC-SSC dot plots of CD4^+^IL-10^+^ and CD4^+^FoxP3^+^ cells were taken from each mouse spleen cell. BTBR and C57 mice were treated with 1250 µg/kg/d of AFB_1_ daily for 28 d via oral administration (p.o.). The control C57 and BTBR mice received saline only via p.o. administration. The statistical analysis was performed using two-way ANOVA followed by Tukey’s post hoc test, which was corrected for multiple comparisons. All data are shown as mean ± SD (*n* = 10/group). * *p* < 0.05, compared to saline-treated C57 mice; ^a^ *p* < 0.05, compared to saline-treated BTBR mice.

On further evaluating the effects of AFB_1_ exposure on the proportions of IL-10-, TGF-β1-, and FoxP3-expressing CD4^+^ T cells, we found significantly lower proportions of these cells in the BTBR mice treated with AFB_1_, as compared to those in the saline-treated BTBR mice (Figure 7A–C): strain [BTBR > C57: F(1,20) = 119.7, *p* = 0.0001], exposure [AFB_1_ > no AFB_1_: F(1,20) = 70.20, *p* = 0.0001], and exposure × strain interaction [F(1,20) = 134.1, *p* = 0.0016] effects, in the case of CD4^+^IL-10^+^ cells; strain [BTBR > C57: F(1,36) = 123.9, *p* = 0.0001], exposure [AFB_1_ > no AFB_1_: F(1,36) = 19.19, *p* = 0.0003], and exposure × strain interaction [F(1,36) = 6.338, *p* = 0.0205] effects, in the case of CD4^+^TGF-β1^+^ cells; and strain [BTBR > C57: F(1,36) = 148.6, *p* = 0.0001], exposure [AFB_1_ > no AFB_1_: F(1,36) = 47.32, *p* = 0.0001], and exposure × strain interaction [F(1,36) = 1.233, *p* = 0.0369] effects, in the case of CD4^+^FoxP3^+^ cells.

Quantitative PCR analyses also demonstrated a decrease in IL-10, TGF-β1, and FoxP3 mRNA expression levels in the brain tissues of the AFB_1_-treated BTBR mice than those of the saline-treated BTBR mice (Figure 7D–F): strain [BTBR > C57: F(1,20) = 81.29, *p* = 0.0001], exposure [AFB_1_ > no AFB_1_: F(1,20) = 11.94, *p* = 0.0025], and exposure × strain interaction [F(1,20) = 1.445, *p* = 0.0016] effects, in the case of IL-10; strain [BTBR > C57: F(1,20) = 77.88, *p* = 0.0001], exposure [AFB_1_ > no AFB_1_: F(1,20) = 15.61, *p* = 0.0008], and exposure × strain interaction [F(1,20) = 1.619, *p* = 0.0033] effects, in the case of TGF-β1; and strain [BTBR > C57: F(1,36) = 92.24, *p* = 0.0001], exposure [AFB_1_ > no AFB_1_: F(1,36) = 20.74, *p* = 0.0002], and exposure × strain interaction [F(1,36) = 6.981, *p* = 0.0156] effects, in the case of FoxP3.

AFB_1_ exposure also significantly downregulated IL-10 and FoxP3 protein expression in BTBR mice, as compared to that found in BTBR mice treated with saline (Figure 7G,H): strain [BTBR > C57: F(1,20) = 60.27, *p* = 0.0001], exposure [AFB_1_ > no AFB_1_: F(1,20) = 15.58, *p* = 0.0008], and exposure × strain interaction [F(1,20) = 22.83, *p* = 0.0004] effects, in the case of IL-10; strain [BTBR > C57: F(1,20) = 95.27, *p* = 0.0001], exposure [AFB_1_ > no AFB_1_: F(1,20) = 31.76, *p* = 0.0001], and exposure × strain interaction [F(1,20) = 18.55, *p* = 0.0013] effects, in the case of FoxP3. Therefore, our results demonstrated that AFB_1_ exposure increases immune abnormalities by downregulating Tregs in BTBR mice.

## 3. Discussion

The effects of AFB_1_ as a toxic immune agent are well known, and exposure to it has been shown to induce immune and pro-inflammatory responses [59,60]. A recent study reported that AFB_1_ aggravates behavioral impairments and oxidative status imbalances and is, thus, potentially toxic [61]. AFB_1_ exposure has also been shown to have detrimental consequences and contribute to the aggravation of medical comorbidities in children with ASD [62]. AFB_1_ induces oxidative stress-mediated microglial cell apoptosis through the NF-κB signaling pathway in the spinal cord [63]. Another study confirmed that AFB_1_ exposure could cause an inflammatory reaction in microglial cells, which is potentially harmful to the CNS and may increase susceptibility to neurodegenerative diseases [51]. In this study, we evaluated the effects of AFB_1_ exposure on autism-like behavior in a BTBR mouse model of autism. BTBR mice treated with AFB_1_ exhibited increased stereotypical, repetitive, and impaired social behaviors. The AFB_1_-exposed BTBR mice displayed social interaction deterioration in the three-chambered sociability test. In addition, the animals also exhibited markedly exacerbated neurobehavioral deficits. Thus, it may be concluded that AFB_1_ exposure can cause behavioral abnormalities associated with social and repetitive behaviors.

Multiple research studies have been conducted to ascertain the potential involvement of immunological function in the etiology of ASD. Elevated concentrations of IFN-γ have been documented to be implicated in the pathogenesis of ASD [55,64]. The brain has been observed to exhibit elevated levels of IFN-γ expression [6]. Previous research has indicated that there is an increase in IFN-γ levels among both children diagnosed with ASD [65] and women who have children with ASD [66]. T-bet plays a crucial function in the progression of diseases and is expressed in T cells that infiltrate the CNS [16,67]. Cells expressing T-bet have been documented to possess encephalitogenic properties within the CNS, and their infiltration is linked to neuroinflammation [68]. T-bet is primarily triggered by the activation of STAT1 [69], which is associated with the production of IFN-γ [70]. The present work demonstrated notable elevations in the proportions of CD4^+^ T cells expressing IFN-γ, STAT1, and T-bet in BTBR mice following treatment with AFB_1_. Furthermore, the animals exhibited a notable elevation in IFN-γ, STAT1, and T-bet expression at both the mRNA and protein levels inside the brain tissue. Hence, the inflammatory consequences of exposure to AFB_1_ can be elucidated by its capacity to enhance the activation of Th1 signaling. The findings of this study suggest that AFB_1_ has the potential to exacerbate autism-like symptoms in BTBR mice through the activation of Th1 signaling.

Recent research investigations have indicated that there is a notable increase in IL-9 levels among children diagnosed with ASD as well as in animal models used to study ASD [14,71]. Furthermore, previous studies have demonstrated the crucial role of IL-9 in activating T cells during inflammation of the CNS [22]. The involvement of IL-9 in brain cells has been postulated [72], specifically with a notable increase in IL-9 production observed in brain pericytes [73]. Patients diagnosed with Rett syndrome have exhibited significantly elevated levels of IL-9 in their serum compared to individuals in the control group [74]. Previous studies have provided evidence indicating that IL-9 is expressed within the CNS [20]. A separate investigation demonstrated that IL-9 regulates macrophage activation within the brains of individuals with progressive MS [21]. IRF4, a constituent of the IRF transcription factor family, has expression in most cells within the immune system [75]. Recent research has provided evidence indicating that the IRF4 gene impacts the neuroinflammation process [75]. The results of our investigation indicate that BTBR mice, when exposed to AFB_1_, exhibited a notable increase in the levels of IL-9 and IRF4 in both spleen cells and brain tissues. The research demonstrates that AFB_1_ triggers the activation of Th9 signaling, leading to immunological dysregulation in BTBR mice. This suggests a potential involvement of AFB_1_ in the immune and behavioral dysfunctions observed in these animals. The findings of this study suggest that exposure to AFB_1_ is associated with an elevated risk of developing ASD.

Prior research has established that Th17 cells have a role in the manifestation of behavioral impairments in individuals with ASD, indicating that inflammation in the peripheral system affects the development of neurons [5,76]. Prior research has demonstrated that the expression of IL-17A is elevated in the immune cells located in the periphery and the brain tissues of BTBR autistic mice and children diagnosed with ASD [9,17]. According to another study, there is a correlation between elevated levels of IL-17A and the severity of symptoms in individuals with ASD [27]. Furthermore, there have been reports indicating that the levels of maternal IL-17A have an impact on the development of ASD-like characteristics in the offspring [30]. Elevated levels of IL-21 have been observed in mice brain damage [77]. Furthermore, it has been observed that children diagnosed with ASD exhibit heightened levels of IL-21 expression [78]. Previous studies have provided evidence indicating a notable increase in the expression of RORγT in children diagnosed with ASD and BTBR mice [14,79]. According to Choi et al., it has been suggested that the prevention of ASD development could be achieved by targeting RORγT signaling [30]. Additionally, evidence supports the notion that suppressing RORγT could be a more efficacious approach to treating neuroinflammation [80]. The pro-inflammatory activities of microglia are mediated by the STAT3 signaling pathway [30]. Activating the STAT3 signaling pathway elicits neuroinflammatory reactions while suppressing STAT3 activity decreases aberrant behavior and neuroinflammation [80,81]. Previous studies have reported increased STAT3 expression in children diagnosed with ASD and BTBR mice [9,14,62]. Our investigation showed that exposure to AFB_1_ increased the quantity of CD4^+^ T cells expressing IL-17A, IL-21, RORγT, and STAT3 in BTBR mice. IL-17A, IL-21, RORγT, and STAT3 expression at the mRNA and protein levels substantially increased in BTBR mice treated with AFB1. This suggests that the neuroinflammatory consequences of AFB_1_ may be attributed to the enhancement of pro-inflammatory signaling pathways in BTBR animals. This study is the initial report documenting the upregulation of Th17 signaling due to AFB_1_ exposure, perhaps linked to behavioral and neuroimmune abnormalities in BTBR mice. Hence, the impact of exposure to AFB_1_ on Th17 cells may potentially exacerbate neuroimmunological diseases.

IL-22 is known to impact several immune-mediated illnesses significantly, and its expression has been linked to the activation of lymphocytes in the brain [82]. According to a prior investigation, heightened expression of IL-22 has been implicated in facilitating the infiltration of leukocytes into the brain [83]. A separate investigation has documented that elevated levels of IL-22 play a role in the pathogenesis of neurodegenerative diseases [84]. The expression of TNF-α has been observed to be elevated in individuals diagnosed with ASD [55]. A more recent study has indicated a significant correlation between elevated levels of TNF-α and ASD [85,86]. Moreover, evidence suggests that the participation of AhR signaling may be linked to the severity of ASD [87]. The findings of this study indicate that BTBR mice treated with AFB1 exhibited a notable increase in the expression of IL-22, TNF-α, and AhR in CD4^+^ T cells. Additionally, an upregulation was observed in the mRNA and protein expression of IL-22, TNF-α, and AhR. Therefore, it can be inferred that AFB_1_ increases Th22 signaling and disrupts the immunological homeostasis in BTBR mice. Nevertheless, the precise mechanism by which AFB_1_ enhances the activation of Th22 signaling has yet to be determined.

Tregs have been found to have a crucial role in preventing immune-mediated inflammation [88,89]. Immunopathological diseases are mitigated by their pivotal role in modulating the immune system [90]. Recent research has indicated a decrease in Tregs in the brain, peripheral blood, and spleen of an animal model of autism and children diagnosed with ASD [9,14]. Tregs play a crucial role in maintaining self-tolerance through the secretion of the anti-inflammatory cytokine IL-10 [91,92]. Previous research has demonstrated that reduced levels of IL-10 indicate heightened inflammatory states in mice models [93,94]. A separate investigation was conducted to analyze IL-10, a cytokine known for its immunosuppressive properties and association with Treg function [3,95]. The findings of our study indicate a decrease in the expression of IL-10, TGF-β1, and FoxP3 in CD4^+^ T cells in BTBR mice following exposure to AFB_1_. Additionally, it was shown that exposure to AFB_1_ decreased the expression levels of IL-10, TGF-β1, and FoxP3 mRNA and protein in the brain tissue. Hence, the suppression of Treg signaling could potentially play a role in promoting inflammation resulting from exposure to AFB_1_ in BTBR mice. The results of our study indicate that exposure to AFB_1_ may contribute to the development of neuroimmune dysregulation or immunological imbalance in individuals diagnosed with autism disorders. Nevertheless, further data are required to establish a comprehensive link between abnormal immune function and behavior in BTBR mice.

## 4. Conclusions

Our findings demonstrated that exposure to AFB_1_ exacerbated behavioral impairments and immunological dysfunction in BTBR mice. The effects in these animals were attributed to the induction of numerous signaling pathways and the overexpression of inflammatory markers, such as Th1/Th9/Th22/Th17 signaling, which were mediated by AFB_1_ exposure. In general, the deregulation of immune responses, including Th1, Th9, Th17, Th22, and Treg cells and their associated transcription factors, may potentially contribute to the manifestation of ASD-like behavior in BTBR mice. Hence, the outcomes of our study possess the potential to inform the development of enhanced therapeutic interventions aimed at mitigating the social impairments and immunological dysregulation associated with autism following exposure to AFB_1_. The extent of AFB_1_ exposure can exhibit substantial variation across diverse animal species. The findings observed in a BTBR autistic mice model may not have direct translational relevance to the human population. The findings of this study may have limited generalizability.

## 5. Materials and Methods

### 5.1. Chemicals and Antibodies

The following reagents were acquired from Sigma-Aldrich (St. Louis, MO, USA): AFB1, ionomycin, PMA, and RPMI-1640 medium. The fluorescently labeled antibodies targeting IFN-γ, STAT1, T-bet, IL-9, IRF4, IL-17A, IL-21, RORγT, STAT3, IL-22, AhR, TNF-α, IL-10, TGF-β1, FoxP3, and the buffers for red blood cell permeabilization/fixation were procured from BioLegend (San Diego, CA, USA). The Golgi-Plug and RORγT reagents were acquired from BD Biosciences (San Diego, CA, USA). The primary antibodies used in this study, including IFN-γ, T-bet, IL-9, IL-17A, RORγT, TNF-α, IL-10, and FoxP3, were acquired from Santa Cruz Biotechnology (Dallas, TX, USA). The FcR blocking reagent was acquired from Miltenyi Biotech (Bergisch Gladbach, Germany). The nitrocellulose membranes utilized in this study were acquired from Bio-Rad Laboratories (Hercules, CA, USA). The primers utilized in this work were acquired from GenScript (Piscataway, NJ, USA). The Merck, Darmstadt, Germany’s chemiluminescence kit was utilized to conduct Western blotting. The TRIzol^®^ reagent used in this study was obtained from Life Technologies (Carlsbad, CA, USA). The SYBR^®^ Green and cDNA kits utilized in this study were purchased from Applied Biosystems (Foster City, CA, USA).

### 5.2. Animals

Male mice of the BTBR T^+^Itpr3^tf^/J (BTBR) and C57BL/6 (C57) strains, aged 7–8 weeks and weighing 25–30 g, were procured from Jackson Laboratory (Bar Harbor, ME, USA). The mice were housed in a controlled environment, adhering to a 12 h light and 12 h dark cycle at a temperature of 25 °C. This environment was specifically designed to be free from any pathogens. The animals were provided with water and fed standard mouse chow from the Animal Center of the College of Pharmacy at King Saud University in Riyadh, Kingdom of Saudi Arabia. All experimental techniques conducted in this study were authorized by the Institutional Animal Care and Use Committee of King Saud University, with the ethical approval number KSU-SE-22-54.

### 5.3. AFB_1_ Exposure

The mice were allowed to acclimatize for 2–3 weeks and divided into four groups of 6–10 mice each, as follows. AFB_1_ was dissolved in normal saline (0.9% NaCl), and the appropriate concentrations were prepared for all experiments: Group 1—C57 mice treated with saline alone; Group 2—C57 mice orally treated with 1250 µg/kg/d AFB_1_ daily for 28 d; Group 3—BTBR mice treated with saline alone; and Group 4—BTBR mice treated with 1250 µg/kg/d AFB_1_ daily for 28 d. The doses of AFB_1_ were selected based on previous studies [58,96]. Clinical signs of toxicity and death were recorded during the experiment, and all animals tolerated these doses without any toxic symptoms or death. Expert researchers conducted behavioral experiments on days 27 (marble-burying and self-grooming behavior tests) and 28 (social interaction test), followed by animal sacrifice on day 29. Spleen and brain tissues were obtained from these animals for flow cytometry and mRNA and protein expression analyses.

### 5.4. Three-Chambered Social Approach

The three-chamber paradigm was used as previously described [53,97]. Briefly, the apparatus was a non-glare Perspex box (22 × 60 × 22 cm) in which the test mouse was placed and allowed to habituate for 10 min by lifting the left and right retractable doors simultaneously. The test began upon simultaneously removing the left and right retractable doors, separating the central chamber for 10 min, during which the subject mouse could explore all the chambers. Two independent blinded observers recorded social interactions, as previously described [53,97].

### 5.5. Self-Grooming

Mice were scored for self-grooming behavior, as reported previously [52]. After a 10 min habituation period in the test cage, the cumulative amount of time each mouse spent grooming all body regions in 10 min was recorded by a well-trained staff member blinded to the treatment groups. As previously described, the observer sat approximately 2 m from the test cage [53,57].

### 5.6. Marble-Burying Test

Marble-burying was measured in a standard mouse cage with 20 green glass marbles placed at a 5 cm depth of clean bedding, arranged in a 4 × 5 grid, according to a previous method [54,60]. Each mouse was allowed to freely explore and bury the marbles for 30 min. Marbles were considered buried if at least 2/3rd of the marble was covered by bedding, as previously described [54,57,97].

### 5.7. Preparation of Mouse Spleen Cells

Spleens were extracted from different groups, and a single-cell suspension was prepared using the established method [9]. Spleen cells were isolated by smashing the tissue with a stainless-steel mesh in RPMI-1640 medium containing 10% FBS, 50 μM 2-mercaptoethanol, and 1% antibiotic antimycotic solution (Sigma-Aldrich (St. Louis, MO, USA)). The cells were obtained using centrifugation at 300× *g* for 10 min and resuspended in 3 mL red blood cell lysis buffer. After incubation for 10 min at room temperature, the cells were centrifuged at 300× *g* for 10 min and resuspended in RPMI-1640 medium.

### 5.8. Flow Cytometry

Flow cytometric analysis was performed to evaluate the IFN-γ-, STAT1-, T-bet-, IL-9-, IRF4-, IL-17A-, IL-21-, RORγT-, STAT3-, IL-22-, AhR-, TNF-α-, IL-10-, TGF-β1-, and FoxP3-expressing CD4^+^ T cells from spleens. Briefly, splenocytes were incubated with PMA/ionomycin, and Golgi-Plug was added before staining, as previously reported [15]. After washing with washing buffer/PBS, the cells were collected and stained with PE/dazzle-anti-CD4, FITC-anti-CD4, PE-anti-CD4, PE-anti-IFN-γ, PE/cyanine7-anti-STAT1, PE/dazzle-anti-T-bet, PE-anti-IL-9, FITC-anti-IRF4, PE/dazzle-anti-IL-17A, PE-anti-IL-21, PE-anti-RORγT, Alexa Fluor^®^ 647-anti-STAT3, allophycocyanin (APC)-anti-IL-22, PE-anti-AhR, APC/cyanine7-anti-TNF-α, PE/dazzle-anti-IL-10, FITC-anti-TGF-β1, and PE-anti-FoxP3 fluorescent antibodies. Lymphocytes were isolated from other immune cells (monocytes and granulocytes) using a traditional gating technique based on physical qualities (forward and side scatter) to determine the distinct immunological markers in lymphocytes. The immunofluorescence features of antibody-labeled cells in the lymphocyte gate were used to identify cytokines and transcription factors. The proportions of CD4^+^IFN-γ^+^, CD4^+^STAT1^+^, CD4^+^T-bet^+^, CD4^+^IL-9^+^, CD4^+^IRF4^+^, CD4^+^IL-17A^+^, CD4^+^IL-21^+^, CD4^+^RORγT^+^, CD4^+^STAT3^+^, CD4^+^IL-22^+^, CD4^+^AhR^+^, CD4^+^TNF-α^+^, CD4^+^IL-10^+^, CD4^+^TGF-β1^+^, and CD4^+^FoxP3^+^ cells were determined at the lymphocyte gates. Samples were analyzed using a Beckman Coulter FC 500 Flow Cytometer (Beckman Coulter, Indianapolis, IN, USA). All data were analyzed using CXP software (Beckman Coulter).

### 5.9. RT-qPCR

RNA was extracted from the brain tissues using TRIzol^®^ and quantified as previously described [9]. cDNA was synthesized using a cDNA reverse transcription kit, and RT-qPCR analysis was performed using SYBR^®^ Green Master Mix. The primers used in the assay were as follows: *IFN-γ* forward, 5′-AGGAAGCGGAAAAGGAGTCG-3′, and reverse, 5′-GGGTCACTGCAGCTCTGAAT-3′; *STAT1* forward, 5′-TGGGCGTCTATCCTGTGGTA-3′, and reverse, 5′-TGAATGTGATGGCCCCTTCC-3′; *T-bet* forward, 5′-AACAAGGGGGCTTCCAACAA-3′, and reverse, 5′-CCACTGGAAGGATAGGGGGA-3′; *IL-9* forward, 5′-ACCAGCTGCTTGTGTCTCTC-3′, and reverse, 5′-CGGCTTTTCTGCCTTTGCAT-3′; *IRF4* forward, 5′-GGGTGCTTTCTGTTGGCTTG-3′, and reverse, 5′-CTGGCTTGCCAAACACTGTC-3′; *IL-17A* forward, 5′-GGACTCTCCACCGCAATGAA-3′, and reverse, 5′-GGGTTTCTTAGGGGTCAGCC-3′; *RORγ* forward, 5′-AGCTGTGGGGTAGATGGGAT-3′, and reverse, 5′-ATCCGGTCCTCTGCTTCTCT-3′; STAT3 forward, 5′-ATCCTAAGCACAAAGCCCCC-3′, and reverse, 5′-TCCTCACATGGGGGAGGTAG-3′; *IL-22* forward, 5′-GGGGAGAAACTGTTCCGAGG-3′, and reverse, 5′-GGCAGGAAGGAGCAGTTCTT-3′; *TNF-α* forward, 5′-GGACTAGCCAGGAGGGAGAA-3′, and reverse, 5′-CGCGGATCATGCTTTCTGTG -3′; *AhR* forward, 5′-TTCAGAACTGACTCCACCGC-3′, and reverse, 5′-CCGGGTGTGATATCGGGAAG-3′; *IL-10* forward, 5′-CAGAGAAGCATGGCCCAGAA-3′, and reverse, 5′-AGGACACCATAGCAAAGGGC-3′; *TGF-β1* forward, 5′-ACTGCAAGTCAGAGACGTGG-3′, and reverse, 5′-CATAGATGGCGTTGTTGCGG-3′; *FoxP3* forward, 5′-CACAGCAACAGCACTGGAAC-3′, and reverse, 5′-AGCCCTGATGGATGTCTCCT-3′; and *GAPDH* forward, 5′-GGCAAATTCAACGGCACAGT-3′, and reverse, 5′-TGAAGTCGCAGGAGACAACC-3′. Amplification reactions were performed using the 7500 Fast RT-PCR System (Applied Biosystems), and relative changes in gene expression were determined using the 2^−ΔΔCT^ method [98], with GAPDH as the reference gene.

### 5.10. Western Blot Analysis

Proteins were extracted from the mouse brain tissue [99] and quantified using a Direct Detect^®^ infrared spectrometer (Merck). Briefly, 40–50 µg of protein from each sample was separated using 10% SDS-PAGE and transferred to nitrocellulose membranes [15,100]. The membranes were incubated with primary mouse monoclonal antibodies against IFN-γ, T-bet, IL-9, IL-17A, RORγ, IL-22, IL-10, and FoxP3, followed by incubation for two hours with peroxidase-conjugated secondary antibodies at room temperature. The bands corresponding to IFN-γ, T-bet, IL-9, IL-17A, RORγ, IL-22, IL-10, and FoxP3 were visualized using a Western blot detection chemiluminescence kit and quantified relative to the β-actin bands [100].

### 5.11. Statistical Analysis

Data are presented as mean ± SD. Data were analyzed using a two-way analysis of variance followed by Tukey’s post hoc correction for multiple comparisons. All experiments were performed using Prism 5 software (GraphPad, San Diego, CA, USA). *p*-value < 0.05 was considered to be statistically significant.

## Data Availability

All data presented in this study are available on reasonable request from the corresponding author.
